# Antifouling and Desalination Enhancement of Forward Osmosis-Based Thin Film Composite Membranes via Functionalized Multiwalled Carbon Nanotubes Mixed Matrix Polyethersulfone Substrate

**DOI:** 10.3390/membranes15080240

**Published:** 2025-08-08

**Authors:** Hamza E. Almansouri, Mohamed Edokali, Mazrul N. Abu Seman, Ellora Priscille Ndia Ntone, Che Ku Mohammad Faizal Che Ku Yahya, Abdul Wahab Mohammad

**Affiliations:** 1 Faculty of Chemical and Process Engineering Technology, Universiti Malaysia Pahang Al-Sultan Abdullah, Lebuhraya Persiaran Tun Khalil Yaakob, Kuantan 26300, Pahang, Malaysia; ellorapriscille@gmail.com (E.P.N.N.); mfaizal@umpsa.edu.my (C.K.M.F.C.K.Y.); 2 Chemical Engineering Department, Faculty of Engineering, University of Benghazi, Benghazi P.O. Box 1308, Libya; 3 School of Chemical and Process Engineering, University of Leeds, Leeds LS2 9JT, UK; m.edokali.88@outlook.com; 4 Centre for Sustainability of Mineral and Resource Recovery Technology (SMaRRT), Universiti Malaysia Pahang Al-Sultan Abdullah, Lebuh Persiaran Tun Khalil Yaakob, Kuantan 26300, Pahang, Malaysia; 5 Chemical and Water Desalination Program, College of Engineering, University of Sharjah, Sharjah 27272, United Arab Emirates; a.binmohammad@sharjah.ac.ae

**Keywords:** forward osmosis, thin-film composite membranes, multiwalled carbon nanotubes, desalination performance, antifouling properties, internal concentration polarization

## Abstract

The growing scarcity of freshwater worldwide has increased interest in forward osmosis (FO) membranes as a promising solution for water desalination and wastewater treatment. This study investigates the enhancement of thin-film composite (TFC) FO membranes via the incorporation of carboxyl-functionalized multiwalled carbon nanotubes (COOH-MWCNTs) into the polyethersulfone (PES) support layer. The membranes were fabricated using a combination of phase inversion and interfacial polymerization techniques, with COOH-MWCNTs incorporated into the membrane support layers at different concentrations (0–0.75 wt.%). Comprehensive characterization was carried out using various analytical methods and mechanical testing to evaluate the physicochemical and structural properties of the membranes. The modified membranes demonstrated improved hydrophilicity, enhanced mechanical and thermal stability, and improved surface charge properties. Performance tests using a 1 M NaCl draw solution showed that the optimized membrane (0.5 wt.% COOH-MWCNTs) attained a 161% enhancement in water flux (7.48 LMH) compared to the unmodified membrane (2.86 LMH), while also reducing internal concentration polarization (ICP). The antifouling properties were also significantly improved, with a flux recovery rate of 91.92%, attributed to enhanced electrostatic repulsion as well as surface and microstructural modifications. Despite a moderate rise in reverse solute flux, the specific reverse solute flux (J_s_/J_w_) remained within acceptable limits. These findings highlight the potential of COOH-MWCNT-modified membranes in enhancing FO desalination performance, offering a promising option for next-generation water purification technologies.

## 1. Introduction

The increasing global freshwater shortage has driven extensive research into optimizing water resources through the reuse of domestic and industrial wastewater, as well as the desalination of saline water. Although rivers, lakes, seas, and oceans collectively cover nearly 70% of the Earth’s surface, these water sources require appropriate treatment before they can be utilized for drinking purposes [1,2]. Among various desalination and water treatment technologies, membrane processes have emerged as a particularly attractive solution owing to their minimal energy requirements, modest capital investment, and capability to handle temperature-sensitive materials [3,4]. Amongst these membrane-based technologies, forward osmosis (FO) is a relatively innovative and environmentally friendly membrane-based process that has gained significant attention due to its reduced energy demands and reduced fouling compared to reverse osmosis (RO) [5,6]. In contrast to RO, FO operates without the need for hydraulic pressure; instead, it employs natural osmotic pressure as the driving force to transfer clean water from a feed solution to a draw solution through a semi-permeable membrane [7,8].

The thin-film composite (TFC) membrane is the most commonly utilized membrane in FO applications. It typically features a thin, defect-free polyamide (PA) selective layer, supported by an underlying porous structure [9,10,11,12,13]. This design offers excellent water permeability, high solute rejection, mechanical strength, and chemical stability [14]. Numerous studies have focused on enhancing TFC membranes by functionalizing their PA active layer with nanomaterials, significantly improving key performance parameters, including water flux, solute rejection, and fouling resistance [15,16,17]. However, modifications to the support layer, which address internal concentration polarization (ICP), structural parameter (S) and improve overall structural integrity, are even more critical in achieving optimal membrane functionality and long-term performance [9,18]. Therefore, the performance of TFC membranes is significantly dependent on the properties of the support layer, as it provides the foundation for the PA layer formed through the interfacial polymerization (IP) [10,19,20]. Optimizing the molecular design and fabrication parameters of the membrane substrate is, therefore, critical to achieving high FO performance [9,10]. Polysulfone (PSf) and polyether sulfone (PES) membranes are commonly employed as substrates in TFC-FO membranes due to their robust mechanical properties. However, their hydrophobic nature limits effective porosity in water, leading to higher fouling rates and shorter lifespans [2,9,21,22,23]. Efforts to improve the hydrophilicity of PSf/PES membranes include blending them with hydrophilic fillers such as polyvinyl pyrrolidone (PVP), polyethylene glycol (PEG), polyvinyl alcohol (PVA), and polydopamine (pDA), as well as incorporating nanomaterials, including solid and porous nanoparticles and 1D and 2D nanomaterials, to modify membrane substrates. The incorporation of solid nanoparticles (e.g., titanium dioxide (TiO_2_) [24]), porous nanoparticles (e.g., zeolite [25,26]), 1D nanomaterials such as halloysite nanotubes (HNTs) [27], carbon nanofibers (CNFs) [28], and carbon nanotubes (CNTs) [29,30], and 2D nanomaterials such as graphene oxide (GO) [31,32] and layered double hydroxide (LDH) [33] can significantly enhance substrate porosity and hydrophilicity while reducing the S value for TFC-FO membranes [34]. However, agglomeration of solid and porous nanoparticles can compromise active layer integrity, whereas 1D and 2D nanomaterials exhibit superior compatibility with the polymer matrix due to their high aspect ratios [35]. Among these, 1D carbon nanotubes (CNTs) have recently demonstrated significant potential [30]. The introduction of oxygen-containing functional groups in these nanomaterials enhances their hydrophilicity and dispersibility in solvents and polymer matrices, thereby improving membrane performance [36,37]. This enhanced dispersion not only facilitates more uniform integration into membranes but also mitigates potential health and environmental risks. As reported in the literature, pristine CNTs are generally more toxic than chemically functionalized CNTs, primarily due to residual metal catalysts and poor solubility [38]. Therefore, the functionalization of CNTs is essential to minimize environmental hazards by improving dispersion, preventing aggregation, and ensuring robust integration into membrane matrices, ultimately supporting long-term operational stability [39,40].

Carbon nanotube-based membranes, particularly those utilizing multi-walled carbon nanotubes (MWCNTs), have recently emerged as a promising avenue in TFC-FO membrane development [41,42,43,44]. MWCNTs exhibit distinctive characteristics, including high aspect ratios, nanoscale inner diameters, and smooth, hydrophobic graphitic walls, which facilitate the rapid transport of water molecules [43,45]. Despite these advantages, the inherent hydrophobicity of MWCNTs poses challenges in achieving uniform dispersion within polymer matrices. Functionalization of MWCNTs with hydrophilic groups has been shown to address these issues, enabling their effective incorporation into mixed matrix membrane (MMM) supports. In addition, modifying the microstructure of TFC-FO membranes through the incorporation of MWCNTs has been suggested to enhance membrane performance by mitigating ICP and improving antifouling properties. The presence of MWCNTs in the support layer can facilitate more efficient water transport channels, reducing resistance to flow and limiting the accumulation of foulants, thereby contributing to the long-term operational stability of FO membranes [46,47]. Recent studies have demonstrated that MWCNT-blended PES substrates in TFC-FO membranes can enhance membrane hydrophilicity, permeability, and fouling resistance [21,48]. For example, Wang et al. [29] incorporated 2 wt.% carboxylated MWCNTs into PES supports, resulting in TFC membranes with significantly improved porosity and water flux. Similarly, Rastgar et al. [19] demonstrated that modifying PES support layers of TFC-FO membranes with ZnO-SiO2 core-shell nanoparticles significantly enhanced membrane performance. By incorporating a 0.5% optimal concentration of these nanoparticles, the hydrophilicity of the PES support was enhanced, effectively diminishing ICP. The modification transformed the membrane’s sponge-like pores into elongated, finger-like structures, decreasing the structural parameter (S) from 723 nm to 374 nm. This enhancement resulted in an impressive 117% increase in permeate flux compared to unmodified TFC/PES membranes. Another study by Choi et al. [48] introduced TFC MMMs by blending functionalized carbon nanotubes into PES supports. These membranes increased water flux by 72% compared to conventional TFC membranes, while demonstrating superior reverse solute flux selectivity and improved fouling resistance, which is attributed to their negatively charged surface. Enhanced performance has also been observed with membranes incorporating functionalized MWCNTs blended with other materials, such as PVP and TiO_2_ [21]. These combinations improve structural and hydrophilic properties, leading to increased water flux and better selectivity in comparison to unmodified substrates.

Even with these improvements, challenges remain in optimizing the incorporation of nanomaterials into TFC-FO membrane structures [8,9,21,46]. Issues such as the stability of nanomaterial dispersion in polymer matrices, trade-offs between permeability and selectivity, and the scalability of fabrication processes need to be addressed. Identifying and utilizing effective nanomaterials or nanofillers is crucial for advancing high-performance TFC-FO membranes, with a balanced combination of desalination efficiency, antifouling properties, and durability [18,49]. This research gap underscores the need for systematic investigations that assess the combined effects of these materials on the overall performance of membranes, particularly in terms of desalination, fouling resistance, and structural integrity under practical operating conditions.

This study aimed to develop and evaluate FO-based TFC membranes with functionalized MWCNTs integrated into a mixed matrix PES support layer. The research systematically investigated the effect of varying the nanomaterial loadings on the structural, mechanical, and separation properties of the membranes. Advanced characterization techniques, including Fourier-transform infrared spectroscopy (FTIR), X-ray Photoelectron Spectroscopy (XPS), X-ray Diffraction Spectroscopy (XRD), Field emission scanning electron microscopy (FE-SEM), Atomic force microscopy (AFM), Water contact angle (WCA) measurements, and tensile strength testing, were employed to explain the relationship between nanomaterial incorporation and membrane properties. The membrane’s desalination and antifouling performance were further evaluated using a bench-scale FO system with a focus on organic fouling using sodium alginate as a model foulant, besides using real seawater samples for antifouling performance validation. Notably, while previous studies have investigated the impact of MWCNT incorporation on membrane performance, to the best of our knowledge, limited research has comprehensively assessed the desalination and fouling performance across all nanomaterial loadings. This study addresses this gap by systematically evaluating the membrane’s antifouling characteristics at different MWCNT concentrations. Additionally, unlike many existing studies that focus exclusively on either active layer face feed solution (ALFS) or active layer face draw solution (ALDS), this work comprehensively tested membrane performance in both configurations. To ensure accurate and consistent assessment of separation parameters, a two-stage, non-pressurized testing setup was employed. This dual-mode evaluation provides a more complete understanding of the membrane’s functionality under different osmotic conditions, offering valuable insights into its potential applications in diverse water treatment scenarios.

## 2. Materials and Methods

### 2.1. Material

Polyethersulfone (PES, BASF SE, Ludwigshafen, Germany) was used for the membrane support layer. Polyvinylpyrrolidone (PVP, Sigma-Aldrich, St. Louis, MO, USA) served as the pore former, while N-Methyl-2-pyrrolidone (NMP, Sigma Aldrich) was used as the doping solvent. Carboxylated multiwalled carbon nanotubes (COOH-MWCNTs), (diameter: 10−15 nm, length: 10–30 μm, Shanghai Xingtian New Material Technology Ltd., Co., Shanghai, China) were incorporated as nanofillers into the membrane support. For the PA layer, trimesoyl chloride (TMC, purity ~98.0%, Sigma-Aldrich) and m-phenylenediamine (MPD, purity ≥ 98.5%, Sigma-Aldrich) were used as reacting monomers. Pure water (PURELAB Quest, High Wycombe, UK) was used as the solvent for the MPD solution, and n-hexane (purity ≥ 98.5, Merck, Rahway, NJ, USA) served as the solvent for TMC. Sodium alginate (SA, Sigma Aldrich) served as a model organic foulant, dissolved in calcium chloride dihydrate (CaCl_2_·2H_2_O, purity 99.6%, HmbG, Hamburg, Germany) and sodium chloride (NaCl, purity 99.8%, Bendosen, Damansara, Malaysia), which was utilized for the feed solution (FS) in fouling experiments. During membrane FO performance tests, NaCl dissolved in pure water was employed as the draw solution (DS).

### 2.2. Fabrication of Thin-Film Composite (TFC) Membrane

**Figure 1:**
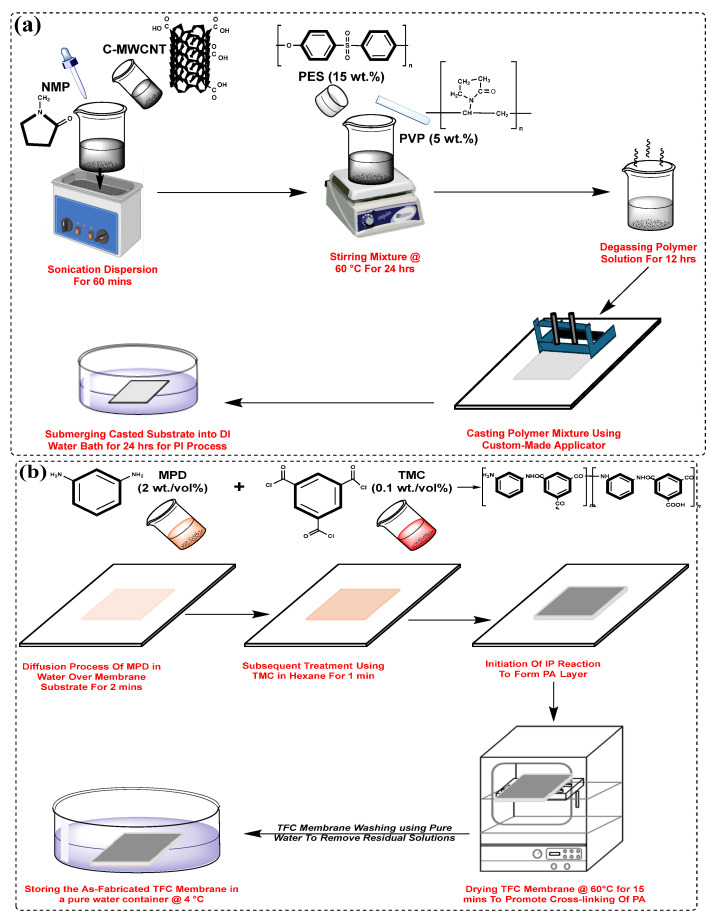
Fabrication process of TFC membranes: (**a**) COOH-MWCNT-Modified PES substrate, and (**b**) PA active layer formed over COOH-MWCNT-Modified support membrane.

In this study, the phase inversion technique was employed to synthesize both unmodified PES and COOH-MWCNTs-modified PES support layers. Firstly, COOH-MWCNTs were dispersed in NMP solvent via sonication for 60 min to ensure uniform distribution. Subsequently, PES (15 wt.%) and PVP (5 wt.%) were incorporated into the solution and subjected to continuous stirring at 60 °C for 24 h. To eliminate air bubbles, the polymer solution underwent degassing for 12 h. The prepared solution was then cast using a custom-designed casting knife to attain an approximate thickness of 200 μm. Immediately after casting, the films were submerged in a deionized (DI) water bath and maintained for 24 h to ensure complete phase inversion (Figure 1a). The resulting support layers served as substrates for the fabrication of TFC membranes. A set of membranes with different COOH-MWCNTs concentrations (0 wt.%, 0.1 wt.%, 0.25 wt.%, 0.5 wt.%, and 0.75 wt.%) was synthesized, as detailed in Table 1.

The PA active layer was fabricated via interfacial polymerization (IP) on top of the support membrane. Initially, a 2 wt./vol% solution of MPD in water was applied to the membrane surface and left for 2 min to allow penetration through the membrane. Following this, any remaining solution was eliminated using a rubber roller. Next, a 0.1 wt./vol % TMC solution in hexane was spread over the surface for 1 min to initiate the IP process. This process resulted in the formation of TFC membranes, which were then heated at 60 °C for 15 min to improve the PA crosslinking [46]. The produced TFC membranes were then rinsed repeatedly with pure water to remove any excess solutions from the surface. Finally, the membranes were preserved in a pure water container at 4 °C for subsequent characterization and evaluation (Figure 1b). Based on the COOH-MWCNTs concentration in the support layer, the membranes were labelled as TFC-0 (control), TFC-1, TFC-2, TFC-3, and TFC-4, respectively.

### 2.3. Characterization

#### 2.3.1. Field Emission Scanning Electron Microscopy (FE-SEM)

The structure of MWCNT nanomaterial, surface, and cross-sectional morphologies of the membranes were observed using FE-SEM (JSM7800F, JEOL, Tokyo, Japan). Prior to FE-SEM imaging, the nanomaterial sample and each membrane sample were carefully mounted onto aluminum SEM stubs and subsequently coated with platinum using an ion sputtering device for 60 s. Images were obtained using normal lens mode with an accelerating voltage of 5 kV. Cross-sectional images of the membranes were also captured after freezing the samples in liquid nitrogen, a step taken to preserve structural integrity during the fracturing process.

#### 2.3.2. Brunauer-Emmett-Teller (BET)

The BET gas adsorption–desorption method, utilizing a Micromeritics Tristar 3020 analyzer (Micromeritics Instrument Corp., Norcross, GA, USA), was employed to investigate the effect of COOH-MWCNT loading on the surface properties of TFC membranes. This technique is essential for characterizing porous materials, providing insights into the surface area, pore volume, and pore size distribution within micro- and mesoporous structures. The samples were analyzed in their solid state after being degassed using a Micromeritics Flow Prep 060 system (Micromeritics Instrument Corp., Norcross, GA, USA). During this process, the samples were heated to 80 °C overnight under a continuous nitrogen (N_2_) gas flow to eliminate moisture and surface contaminants. Following degassing, the samples were weighed to ensure accurate calculations within the analytical software. Subsequently, nitrogen adsorption–desorption measurements were conducted at −196.15 °C to generate adsorption–desorption isotherms, enabling a detailed assessment of the membrane’s porosity characteristics.

#### 2.3.3. Attenuated Total Reflectance Fourier-Transform Infrared (ATR-FTIR) Analysis

ATR-FTIR analysis was performed using a Nicolet iS50 spectrometer (Thermo Fisher Scientific, Waltham, MA, USA) to identify the functional groups present on the MWCNT nanomaterial, as well as on the surface of the fabricated support layers and TFC membranes. Data were recorded in transmission mode over a wavelength range of 500 to 4000 cm^−1^. Each membrane sample underwent 32 scans with a spectral resolution of 4 cm^−1^.

#### 2.3.4. X-Ray Photoelectron Spectroscopy (XPS)

XPS was employed to analyze the elemental composition of MWCNTs, as well as the surface chemistry and chemical structures of the synthesized membranes. The measurements were conducted using a PHI 5000 VersaProbe II system (ULVAC-PHI, Kanagawa, Japan) at two penetration levels: a survey scan and a narrow scan. A monochromatic Al Kα X-ray source (1486.6 eV) was utilized, with a pass energy of 117.4 eV to ensure precise measurements.

#### 2.3.5. X-Ray Diffraction Spectroscopy (XRD)

The crystal structure and atomic spacing of the MWCNTs nanofiller were evaluated using XRD spectroscopy. Measurements were performed with a PANalytical X’Pert^3^ Powder diffractometer (Malvern Panalytical, Almelo, The Netherlands), which was equipped with a Cu-Kα radiation source (λ = 1.5406 − 1.5444 Å). The instrument operated at a fixed voltage of 45 kV and a current of 40 mA. Each scan was conducted with K-A2/K-A1 ratio of 0.5. XRD data were obtained over a 2θ range from 3.0191° to 79.9691°, with a step resolution of 0.05°. Each scan required approximately 29.84 s to complete.

#### 2.3.6. Water Contact Angle (WCA)

The membrane surface wettability was determined using WCA measurements obtained through goniometry (OCA 15EC, DATAPHYSICS, Filderstadt, Germany) via the static sessile drop method. A 5 μL droplet of deionized (DI) water was carefully dispensed onto the membrane surface for analysis. To ensure accuracy and consistency, each measurement was carried out at three distinct points on the membrane, enabling a comprehensive evaluation of droplet stability and reproducibility.

#### 2.3.7. Mechanical Property

The mechanical properties of the membrane support were assessed following the ASTM D882-12 standard [50] using a CT3 Texture Analyzer (Brookfield Engineering, Middleborough, MA, USA). Rectangular membrane samples measuring 50 mm × 10 mm were prepared, and the thickness was measured with a digital micrometer to calculate the cross-sectional area (A). The test was conducted at ambient temperature with a crosshead speed of 2 mm/min and a clamp distance of 30 mm. Each sample underwent a minimum of three repetitions to measure the tensile strength (MPa) and elongation at break (E%). Prior to characterization, all membrane samples were subjected to drying in an oven for 24 h.

#### 2.3.8. Thermal Stability

The thermal stability of membrane supports was conducted using thermogravimetric analysis (TGA) with a Hitachi/ STA7000 instrument (Hitachi High-Tech, Tokyo, Japan). The mass loss of the supports was recorded as the temperature increased from room temperature to 700 °C under a nitrogen (N_2_) at a flow rate of 50 mL/min. The heating rate was controlled at 20 °C/min.

#### 2.3.9. Zeta Potential Measurement

The surface charge of the TFC membranes was analyzed by measuring the zeta potential (ZP) using a Malvern Zetasizer Nano ZSP (Malvern Panalytical, Malvern, UK). Before conducting the ZP measurements, dry membrane samples were placed in a 1 mM KCl solution at 24 °C, with the solution pH (7). Multiple measurements were performed to ensure the stability and reliability of the ZP values for the membrane surfaces. Meanwhile, the ZP of a model foulant solution (500 ppm SA, 50 mM NaCl, and 4 mM CaCl_2_) was measured using a DelsaMax PRO laser photometer (BECKMAN COULTER, Brea, CA, USA) at pH 7 and 24 °C.

#### 2.3.10. Atomic Force Microscopy (AFM)

AFM was used to analyze the surface topography of the TFC membranes using a Park System NX-10 model (Park Systems, Suwon, Republic of South Korea). A key surface topography parameter, average surface roughness (Ra), was quantified. Measurements were conducted in a contact mode over a scanned area of 10 μm × 10 μm.

### 2.4. Determination of Porosity and Pore Size of Membrane Support

Membrane porosity (ε) was measured through the gravimetric method. Circular membrane substrate samples, with diameters of approximately 50 mm, were soaked in water at ambient temperature for 48 h. Excess water was gently wiped off the substrate surfaces, and the wet samples were weighed (W_w_, kg). The samples were afterwards dried overnight in an oven at 60 °C and reweighed (W_d_, kg). The membrane porosity was evaluated by calculating the weight difference prior to and following drying, using the following Equation [28,51]:

(1) $$\varepsilon (\%)=\frac{W_{w}-W_{d}}{A\times l\times \rho_{H2O}}$$

where $\rho_{H2O}$ is the density of water (1000 kg/m^3^), A is an effective area of membrane (m^2^), and l is a membrane thickness (m) measured at multiple separate locations using a micro digital meter.

The mean pore size (rₘ) was calculated using the Guerout-Elford-Ferry equation, which relates the pure water flux (J), and membrane porosity (ε) as follows [52,53]:

(2) $$r_{m}=\sqrt{\frac{(2.9-1.75\varepsilon )\times 8\eta lJ}{\varepsilon \times \Delta P}}$$

Here, η represents the viscosity of water ($8.9\times 10^{-4}Pa\cdot s$), and ΔP denotes the operational pressure (≈1 bar). The pure water flux (J, m/s) of the membrane support was determined using a dead-end filtration system. The experiment was conducted with a stirred cell (HP4750, Sterlitech, Auburn, WA, USA) using pure water as the filtration medium. The membrane substrate was positioned in a membrane cell with an effective area of 14.2 cm^2^. The stirred cell was filled with 100 mL of pure water, which was circulated under an applied pressure of ≈1 bar. The time required for a fixed volume (40 mL) of water to permeate through the membrane was recorded. The pure water flux (J) was determined by dividing the amount of permeate collected within a given time by the active cell area. The results reported represent the average of at least five measurements.

### 2.5. FO Experiments

**Figure 2:**
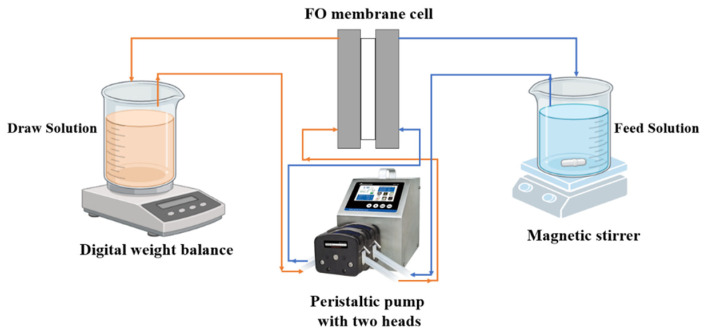
Diagram of the FO lab setup.

The performance of the prepared TFC-FO membranes was assessed using a bench-scale FO system. The assembled setup included a crossflow FO cell with an effective membrane area (A_m_) of 42 cm^2^ (CF042 Cell, Sterlitech, Auburn, WA, USA), a two-headed peristaltic pump (BT600-2J, Longer-Pump, Baoding, China), two 1000 mL tanks for feed and draw solutions, and a weight balance (FX-300i, A&D CO. Ltd., Tokyo, Japan), as depicted in Figure 2. The feed and draw solutions were circulated at a flow rate of 250 mL/min, and the temperatures of both streams were maintained at ambient conditions throughout the experiments.

The water flux (J_w_) was determined by positioning a digital weight balance under the draw solution (DS) tank to measure variations in weight. For reverse solute flux (J_s_), a conductivity meter (Eutech PC 2700, Eutech Instruments Pte. Ltd., Singapore) was employed to monitor the conductivity of the feed solution (FS). A magnetic stirrer was positioned at the base of the feed solution tank to ensure homogeneous mixing of salt during operation. All fabricated membranes were evaluated under two distinct operational configurations: one where the active layer faced the feed solution (ALFS mode) and another where it was oriented towards the draw solution (ALDS mode). Before measurements, the system was allowed to stabilize for 5 min, followed by a 1-h performance test. Each experiment was repeated twice to ensure reproducibility and provide average results. In the FO experiments, pure water was employed as the FS, while the DS was prepared by varying NaCl concentrations from 0.25 to 1.0 M. The water flux (J_w_, LMH) was derived from the weight difference of the draw solution (Δm, g) during the test duration (Δt, h) using the following equation [54,55]:

(3) $$J_{w}=\frac{∆m}{\rho_{H2O}\times A_{m}\times ∆t}$$

Reverse solute diffusion (J_s_, gMH) was computed by determining the salt content in the feed solution (ΔC_t_, g/L). Conductivity measurements were taken and transformed into concentration (M) by utilizing a calibration curve correlating conductivity to salt concentration. The value of J_s_ was then calculated using the following equation [54,55]:

(4) $$J_{s}=\frac{\Delta (C_{t}V_{t})}{A_{m}\times ∆t}$$

To evaluate the performance of the prepared membranes in the FO process, especially with respect to selectivity, the specific reverse solute flux (SRSF, g/L) is a commonly used metric. This measure is calculated by dividing J_s_ by J_w_, providing an essential indication of how efficiently the FO membrane performs. The SRSF was determined through the following equation [56,57]:

(5) $$SRSF=\frac{J_{s}}{J_{w}}$$

Solute rejection (R, %) was evaluated under ALDS mode using a 0.25 M NaCl solution by measuring the salt concentrations on both the permeate (C_p_) and feed (C_f_) sides. The solute rejection percentage (R%) was then calculated as follows [2,54]:

(6) $$R\%=\left(1-\frac{C_{p}}{C_{f}}\right)\times 100$$

### 2.6. Evaluation of Membrane Separation Parameters

The separation parameters of the membrane, including water permeability (A, LMH/bar), solute permeability (B, LMH/bar), and structural parameter (S, µm), were determined using a two-stage non-pressurized method as outlined by Kim et al. [58].

In the first stage, the membrane was positioned in ALDS mode, utilizing four varying concentrations of NaCl solution (0.25, 0.5, 0.75, and 1.0 M) as the DS, while pure water served as the FS. Parameters A and B were calculated from four independent readings of J_w_ and J_s_ using the subsequent equations [58,59,60]:

(7) $$A=\frac{J_{w}}{\pi_{D,b}exp\left(-\frac{J_{w}}{k}\right)-\pi_{F.b}}$$

(8) $$B=\frac{J_{s}}{C_{D,b}exp\left(-\frac{J_{w}}{k}\right)-C_{F,b}exp\left(\frac{J_{w}}{k}\right)}$$

where $\pi_{D,b}$, $\pi_{F.b}$, C_D,b_ and C_F,b_ are the bulk osmotic pressure and bulk concentration of the DS and FS respectively, and k is a mass transfer coefficient. The term $exp\left(-\frac{J_{w}}{k}\right)$ and $exp\left(\frac{J_{w}}{k}\right)$ account for the effects of dilutive external concentration polarization (ECP) and concentrative ICP with the latter being neglected, since pure water was used as the FS. The value of A was derived from the slope of the plot of J_w_ versus ${(\pi }_{D,b}exp\left(-\frac{J_{w}}{k}\right)-\pi_{F.b})$. While the B parameter was obtained as a slope by plotting the graph of Js against the [$C_{D,b}exp\left(-\frac{J_{w}}{k}\right)$].

In the second stage, the S parameter was evaluated with the membrane oriented under ALFS, utilizing the same range of NaCl concentrations as in the first stage for the DS, while pure water was employed as the FS. During this process, the J_w_ values were recorded. These J_w_ values, together with the permeability parameters (A and B) obtained from the first stage, were applied to Equation (9). The S parameter was then calculated numerically by minimizing the global error in Equation (10), using A, B, and S as regression variables [58,59,60,61,62]:

(9) $$J_{w}=A\left[\frac{\pi_{D,b}exp\left(-\frac{J_{w}S}{D}\right)-\pi_{F,b}exp\left(\frac{J_{w}}{k}\right)}{1+\frac{B}{J_{w}}\left[exp\left(\frac{J_{w}}{k}\right)-exp\left(-\frac{J_{w}S}{D}\right)\right]}\right]$$

(10) $$E_{w}=\frac{1}{n}\sum_{i=1}^{n}\sqrt{{\left(1-\frac{J_{w,i}^{CALC}}{J_{w,i}^{EXP}}\right)}^{2}}$$

where D represents the NaCl diffusion coefficient ($1.61\times 10^{-9}m^{2}/s$), which was applied in this study [63].

### 2.7. Fouling Testing Protocol

The fouling resistance of the fabricated membranes was assessed by performing fouling tests within the same crossflow system used for the FO experiments. Sodium alginate (SA) was employed as the foulant agent to investigate membrane fouling under the ALFS mode. Initially, the FO system was stabilized with the FS containing 4 mM CaCl_2_ and 50 mM NaCl, while a 2.0 M NaCl solution served as the DS. This setup was pumped into the FO lab-scale process for 1.5 h to achieve flux stabilization. After stabilization, 500 ppm SA was introduced into the feed solution to initiate the fouling tests. These tests were carried out for 10 h at 25 °C with a flow rate of 250 mL/min. Following each fouling experiment, physical cleaning was performed by circulating pure water counter-currently throughout both sides of the membrane system for 30 min with a flow rate of 250 mL/min. Following this cleaning procedure, the water flux was reassessed under the initial conditions. To further evaluate the anti-fouling performance of the membranes, the flux recovery rate (FRR, %) and total flux decline ratio (Rt, %) were determined as follows [64,65,66]:

(11) $$FRR=\frac{J_{R}}{J_{0}}\times 100$$

(12) $$R_{t}=1-\frac{J_{f}}{J_{0}}\times 100$$

where J_R_ (LMH) represents the recovery flux following membrane cleaning, J_0_ (LMH) stands for the initial water flux, and J_f_ (LMH) denotes the water flux after fouling tests.

## 3. Results and Discussion

### 3.1. Characterization of COOH-MWCNTs

**Figure 3:**
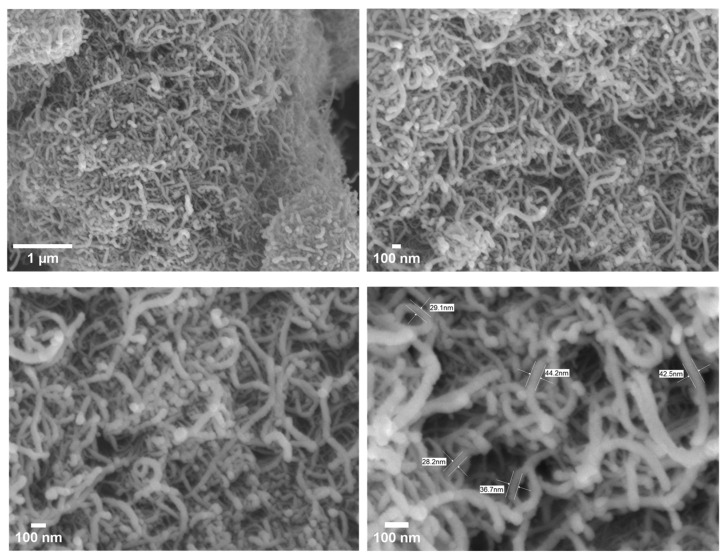
FE-SEM images of COOH-MWCNTs with different magnifications (20 k, 30 k, 50 k, 80 k).

**Figure 4:**
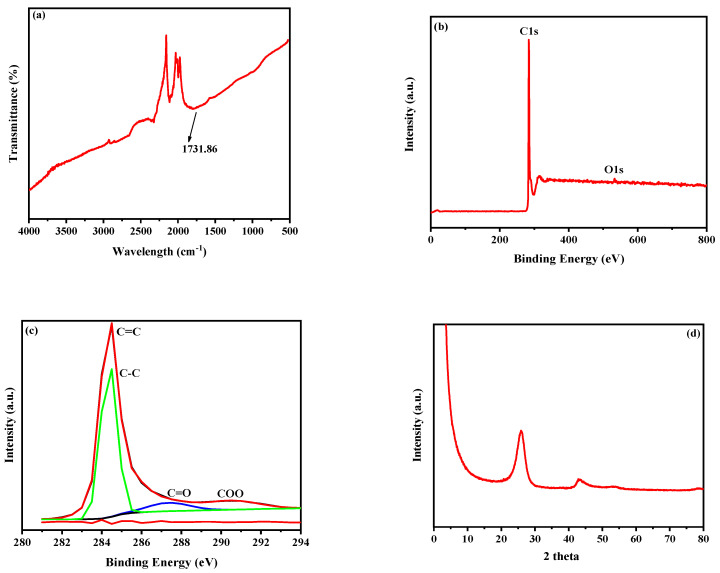
Physicochemical characterizations of COOH-MWCNTs (**a**) FTIR, (**b**) XPS survey scan, (**c**) XPS spectra corresponding to the carbon (C1s) (**d**) XRD pattern.

Figure 3 illustrates the FE-SEM images of COOH-MWCNTs, revealing that they appear as bundles, with surfaces characterized by a long length and a smaller diameter. Additionally, the textural properties of COOH-MWCNTs were characterized using BET surface area, pore volume, and pore size measurements. A high BET surface area of 250.64 ± 7.5 m^2^/g indicates a porous structure that may enhance the hydrophilicity and water permeability of TFC-FO membranes, thereby improving water transport and reducing mass transfer resistance, depending on the material’s loading. Furthermore, with a substantial pore volume of 1.29 ± 0.038 cm^3^/g and a pore size of 20.59 ± 0.62 nm, COOH-MWCNTs are classified as mesoporous, offering an optimal balance between surface area and pore connectivity [67,68]. This structure may support efficient water passage while limiting the accumulation of fouling agents, making COOH-MWCNTs a promising material for water treatment applications. FTIR analysis in Figure 4a shows that carboxylate MWCNTs exhibit a peak at 1731.86 cm^−1^, characteristic of C=O stretching in carboxylic acids [36,69]. The XPS analyses presented in Figure 4b,c were also employed to quantify the elemental composition of COOH-MWCNTs. Prominent peaks for C1s and O1s appear at 284 eV and 533 eV, respectively [70,71], as illustrated in Figure 4b. Furthermore, Figure 4c provides a detailed analysis of the C1s spectra, where COOH-MWCNTs exhibit four binding energy peaks corresponding to C=C (284.36 eV), C-C (284.85 eV), C=O (287.35 eV), and COO (290.46 eV) [72]. Figure 4d shows the XRD pattern of COOH-MWCNTs, which displays two distinct peaks at 25.7° (d-spacing of 0.346 nm), attributed to the distance between the walls in COOH-MWCNTs, and 43.70° (d-spacing of 0.2 nm), corresponding to the inter-wall spacing [73].

### 3.2. Characterization of Membrane Support

**Figure 5:**
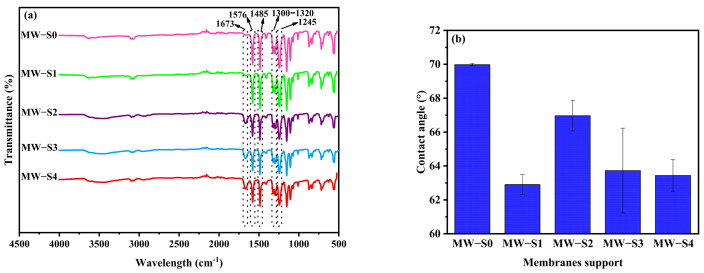
(**a**) FTIR spectra, and (**b**) WCA measurements of membrane supports.

Figure 5a demonstrates FTIR spectra of all fabricated membrane supports. For the control membrane support (MW-S0), spectral peaks were observed at 1576, 1485, 1300 to 1320, and 1245 cm^−1^. These corresponded to the benzene ring, C-C bonds, O=S=O stretching vibrations, and C-O bonds, respectively, highlighting the functional groups of the PES polymer membrane support [19,74,75]. Notably, a new peak appeared at 1673 cm^−1^, which is related to carbonyl stretching in carboxylic acids after blending COOH-MWCNTs with the PES [30,76]. The hydrophilic functional groups of COOH-MWCNTs enhanced the hydrophilicity of the MWCNTs-functionalized PES support, as depicted in Figure 5b. Based on Figure 5b, it was evident that the water contact angle reduced from approximately 70° in the MW-S0 support to 62.88° in the MW-S1 support. Table 2 illustrates the impact of varying amounts of COOH-MWCNTs on the average pore diameter, total porosity, and thickness of membrane supports. Generally, the control support (MW-S0) exhibited a larger pore diameter compared to those integrated with COOH-MWCNTs. Additionally, a higher concentration of COOH-MWCNTs (0.75 wt.%) appeared to increase the average pore size, making it slightly greater than that of the membrane support with a 0.1 wt.% loading (MW-S1). In contrast, smaller pores were achieved with 0.25 and 0.5 wt.% loadings. The modified membrane supports exhibited enhanced porosity relative to the control membrane support, with the highest porosity observed in the membrane support MW-S3 (0.5 wt.%). This suggested that adding appropriate concentrations of COOH-MWCNTs could improve the pathways through the porous support, potentially enhancing the FO performance of membranes [29].

**Figure 6:**
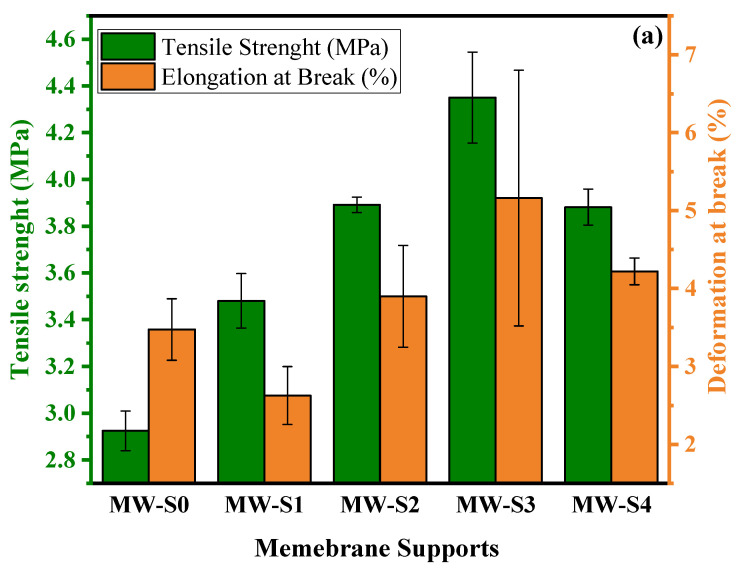

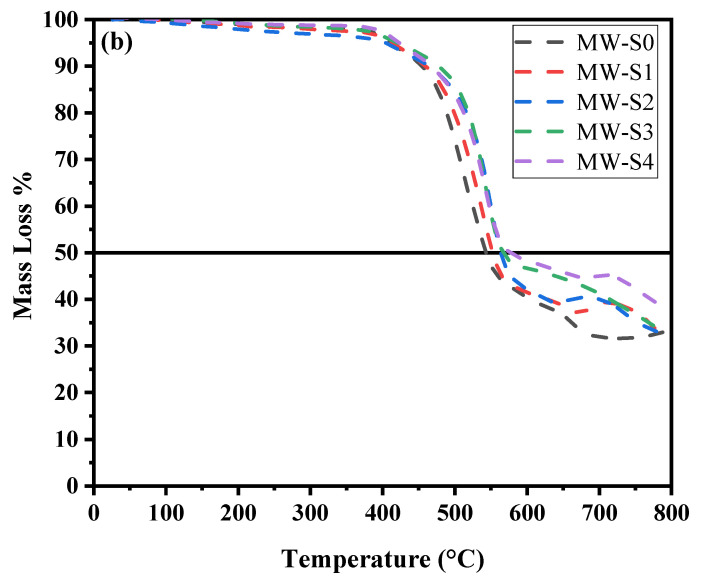
(**a**) Mechanical properties and (**b**) thermal properties of membrane supports.

Figure 6a,b depict the mechanical and thermal characteristics of membrane supports with different COOH-MWCNTs concentrations. The mechanical strength of the membrane supports gradually improved as the COOH-MWCNTs content increased up to 0.5 wt.%. This enhancement can be attributed to the exceptionally high aspect ratio and low density of the 1-D carbon nanotubes [77,78]. However, excessive concentrations of carbon nanotubes may lead to stress concentration sites owing to their aggregation within the membrane matrix [79,80]. In addition, the elongation at break initially declined at 0.1 wt.% but subsequently increased with further additions of COOH-MWCNTs up to 0.5 wt.%. This behavior is owing to enhanced viscoelastic deformation of the PES/COOH-MWCNTs support layer, resulting from increased interactions between the carbon nanotubes and polymer chains [81]. This improvement in mechanical properties indicated that employing COOH-MWCNTs as reinforcing agents in PES was advantageous for creating substrates without fabric [29]. The thermal stability of the membrane supports was assessed through TGA analysis, as shown in Figure 6b. The weight loss pattern was similar for all membrane supports, with mass reduction occurring approximately 500 °C, corresponding to the decomposition temperature of the PES polymer [82,83]. The temperatures at which a 50% mass loss was observed for MW-S0, MW-S1, MW-S2, MW-S3, and MW-S4, were 546 °C, 552 °C, 563 °C, 568 °C, and 576 °C, respectively. These findings indicate that the incorporation of COOH-MWCNTs progressively enhanced the thermal stability of the membrane supports. The enhancement in the mechanical and thermal properties of the membrane support can be attributed to the strong sp^2^ bonds between carbon atoms in carbon nanotubes, which form a robust, self-supporting atomic structure with outstanding thermal stability and exceptional tensile strength along the axial direction [78].

**Figure 7:**
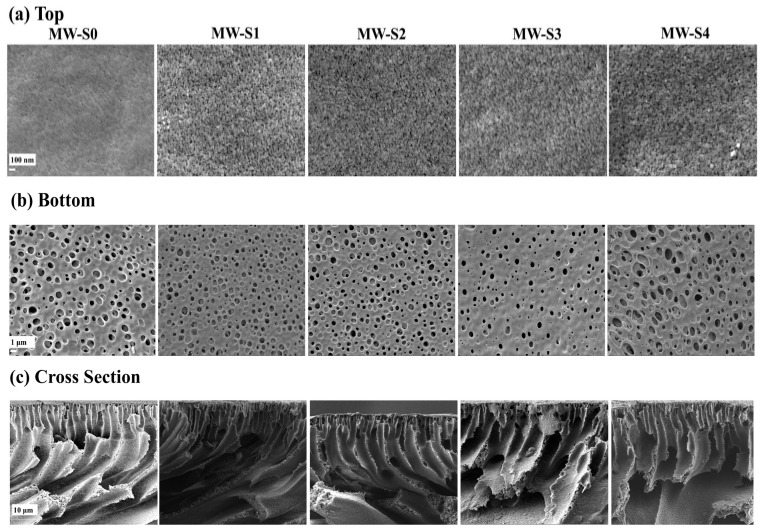
FE-SEM images of the top (**a**) and bottom (**b**) surfaces, and cross-sections (**c**) of the support layers.

The morphologies of the control PES and COOH-MWCNTs-blended PES membranes were studied using FE-SEM micrographs, which displayed the top and bottom surfaces, along with the cross-sections, as illustrated in Figure 7. From the top surface images (Figure 7a), it was evident that varying amounts of COOH-MWCNTs increased the surface porosity of PES-modified membrane supports compared to the PES controls. Adding hydrophilic COOH-MWCNTs to the casting solution enabled them to migrate toward the coagulation bath (water), thereby improving phase separation [84,85]. This, in turn, enhanced both the surface and overall porosity of the modified membrane supports relative to the control membrane, as shown in Table 2. Notably, the top surface of the substrates exhibited significantly smaller pores than the bottom surfaces, which was likely due to the skin layer formed during phase inversion (Figure 7b) [24]. Figure 7c also presents FE-SEM cross-sectional images of all the fabricated membrane supports. It can be observed that the modified support layers have COOH-MWCNTs uniformly distributed within the polymer matrix, with noticeable formation of finger-like macro-voids throughout their structures. In addition, the MW-S0 and MW-S1 modified supports featured finger-like pores near the surface, which expanded into macro-voids towards the base of the membrane. Specifically, MW-S0 had shorter finger-like formations near the surface with larger macro-voids at the bottom, while MW-S1 displayed fewer macro-voids and longer finger-like structures compared to MW-S0. In contrast, the MW-S2 support exhibited elongated finger-like voids, which perpendicularly extended from the top to the bottom of the support layer with slightly developed macro-voids in both depth and width. Meanwhile, the MW-S3 and MW-S4 supports were characterized by long finger-like structures, with noticeably larger voids at the base of MW-S4. Overall, the modified support layers demonstrated progressively developing finger-like structures as the concentration of COOH-MWCNTs increased, which was attributed to the effect of hydrophilic COOH-MWCNTs on the phase separation process [29,85]. This preferred microstructure, with its porous morphology and low tortuosity, can be beneficial for FO membranes by minimizing the structure parameter [29,86,87].

### 3.3. Characterization of TFC Membranes

**Figure 8:**
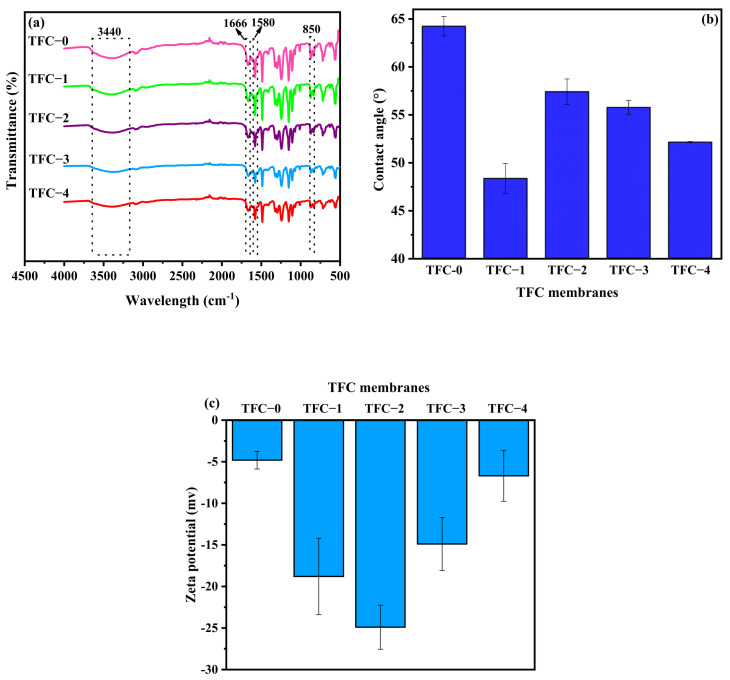
(**a**) FTIR spectra, (**b**) water contact angle, and (**c**) surface zeta potential of TFC membranes.

The ATR-FTIR spectra for the fabricated TFC membranes are illustrated in Figure 8a. As can be seen, notable peaks at 3440 cm^−1^, 1580 cm^−1^, and 1666 cm^−1^ were identified, corresponding to amine II (N-H stretching), amide II (N-H bending), and amide I (C=O stretching), respectively, in both the control and modified TFC membranes [57,88,89]. Furthermore, a distinguishable peak at 850 cm^−1^ was ascribed to the C-Cl stretching vibration of the unreacted acyl chloride group [88]. The presence of N as the primary component for the PA confirmed the successful formation of the PA layer onto the top of the PES support [90]. Figure 8b illustrates the surface hydrophilicity of the fabricated membranes by assessing their water contact angles. The findings revealed that the hydrophilicity of the modified TFC membranes was enhanced compared to the control membrane. Specifically, the membrane with 0.1 wt.% COOH-MWCNTs incorporated into the membrane support (TFC-1) demonstrated the lowest contact angle at approximately 48.33°, as opposed to approximately 64.23° for the control membrane (TFC-0). These results verified that incorporating COOH-MWCNTs into the dope solution enhanced the overall hydrophilicity of the TFC-FO membranes, which can improve its water permeability.

Furthermore, the functionalization of the membrane support with COOH-MWCNTs can modify the physicochemical characteristics of the resulting PA layer. For example, significant differences in surface zeta potential (ZP) were observed between the control and modified TFC membranes, as shown in Figure 8c. All membranes exhibited negatively charged surfaces at a pH of 7.0, attributed to the presence of negatively charged amide groups [91]. The introduction of COOH-MWCNTs into the support layer increased ZP values, altering the surface charges of the PA-TFC membrane to −24.9 mV, −18.8 mV, −14.9 mV, and −6.69 mV for TFC-2, TFC-1, TFC-3, and TFC-4, respectively, whereas the TFC-0 membrane showed the lowest surface charge with approximately −4.81 mV. It can also be noted that the incorporation of COOH-MWCNTs into the membrane support strongly impacted the surface chemistry and the degree of cross-linking in the PA layer, as evidenced by XPS results in Table 3. Accordingly, a low O/N ratio of 1.19 in the TFC-0 signified a highly cross-linked structure, characteristic of dense polyamide networks. Conversely, increased O/N ratios, reaching up to 5.48 in the modified membranes, suggested disrupted or reduced cross-linking due to the incorporation of COOH-MWCNTs into the support layer, resulting in a more linear or loosely cross-linked structure [92,93,94]. Notably, the observed rise in the oxygen content (O%) in the modified TFC membranes, resulting from the incorporation of COOH-MWCNTs, introduced oxygen-rich carboxyl (-COOH) groups into the support layer, increasing surface polarity and enhancing water affinity. Nevertheless, this may hinder the diffusion or availability of MPD at the reaction interface, as MPD monomers could penetrate more deeply into the substrate [2,51]. This finding could potentially reduce the completeness of reactions between MPD and TMC, resulting in fewer amide bonds (-CONH-) and a less cross-linked PA layer that, however, formed a thinner rejection layer, which is favorable for promoting water flux [2,95].

**Figure 9:**
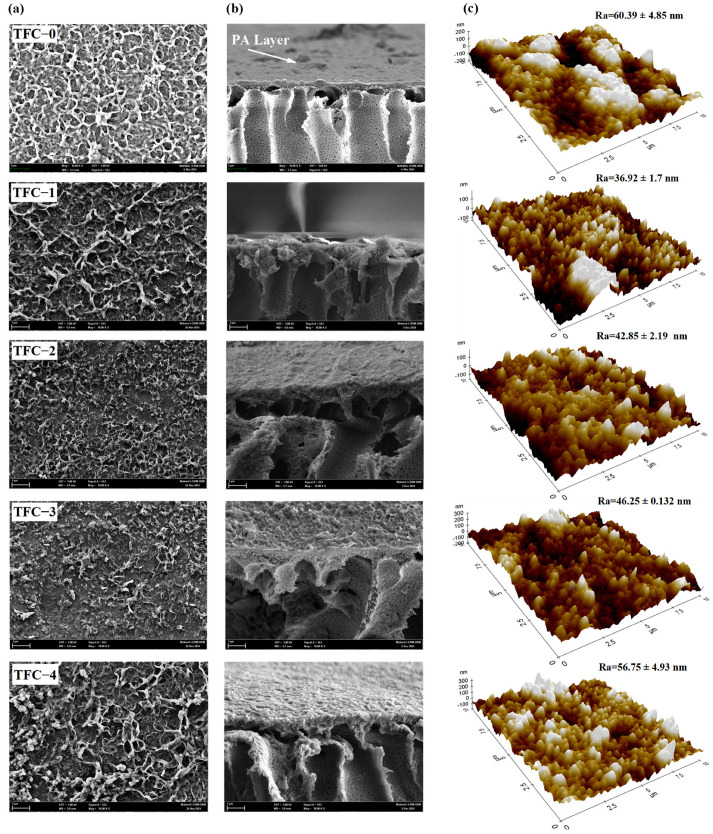
FE-SEM images top (**a**), cross-sectional (**b**), and 3D-AFM images (**c**) of the fabricated PA-TFC FO membranes.

The surface topography and roughness characteristics of the TFC membranes were examined using FE-SEM and AFM, as shown in Figure 9. The FE-SEM micrographs of the synthesized membranes revealed uneven surfaces with a nodular structure [96]. Concurrently, the 3D-AFM images displayed a “ridge-valley” configuration across the membrane surface. This morphological structure resulted from the formation of the PA layer due to the reaction between amine and acyl chloride monomers [97,98]. It was evident that incorporating COOH-MWCNTs into the membrane supports affected membrane roughness. The Ra values of the fabricated membranes decreased from 60.39 nm for TFC-0 to 36.92 nm for TFC-1. Moreover, the roughness values subsequently increased with higher COOH-MWCNTs loadings, following the order TFC-2 < TFC-3 < TFC-4, with values of 42.85 nm, 46.25 nm, and 56.75 nm, respectively.

### 3.4. Effect of COOH-MWCNTs Loading on FO Performance

**Figure 10:**
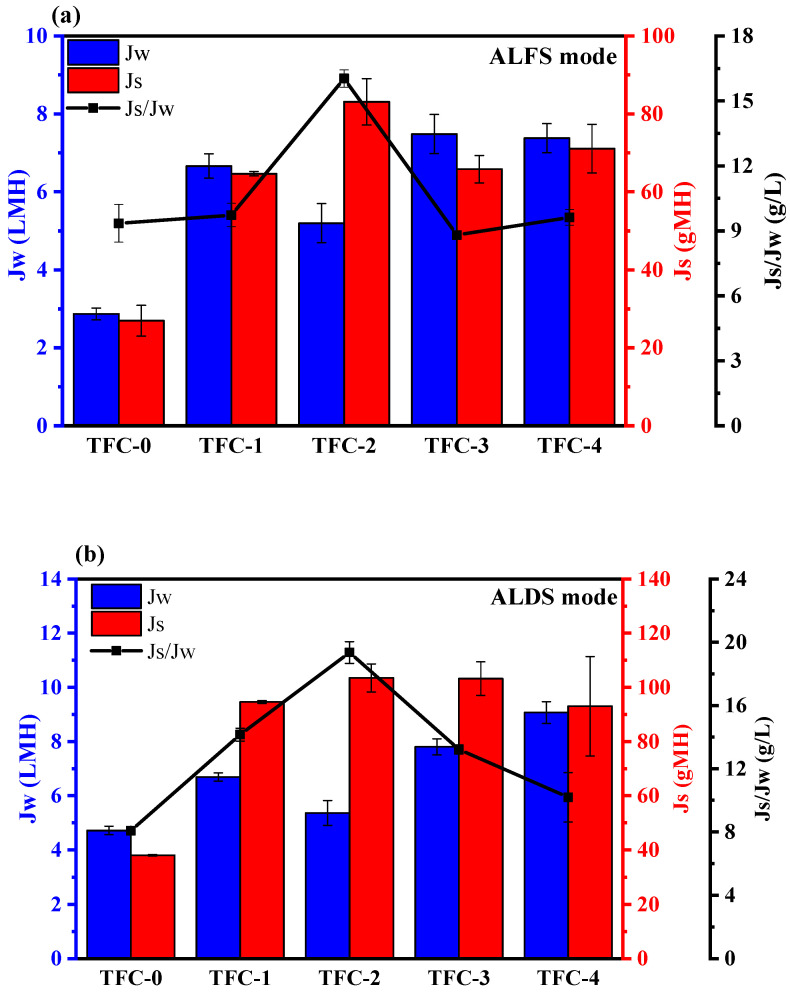
Performance of TFC-FO membranes (i.e., water flux, reverse solute flux, and specific solute flux) operated in (**a**) ALFS mode and (**b**) ALDS mode. Test conditions: [FS = pure water, DS =1.0 M NaCl; Q = 250 mL/min; T = 25 ± 2 °C, Am= 42 cm^2^].

A crossflow FO system was utilized to evaluate the J_w_ and J_s_ of the fabricated TFC membranes. Figure 10a,b present the performance of control and modified TFC membranes, assessed in terms of J_w_, J_s_, and J_s_/J_w_, under ALFS and ALDS configurations using pure water as the FS and 1.0 M NaCl as the DS. Figure S1 additionally shows the performance of all membranes, evaluated by varying NaCl concentrations in the DS from 0.25 M to 1.0 M.

As depicted in Figure 10a,b, all modified membranes, tested in ALFS and ALDS modes, achieved greater water flux than the control membrane. As evident from Figure 10a, the J_w_ exhibited a substantial increase from 2.86 LMH for TFC-0 to 7.48 LMH for TFC-3 (with 0.5 wt.% COOH-MWCNTs), which was slightly higher than that of TFC-4 (with 0.75 wt.% COOH-MWCNTs) at 7.37 LMH; the reduction seen in TFC-4 could be due to the agglomeration of COOH-MWCNTs [99]. The enhancement in water flux under ALFS was likely due to the reduced ICP phenomenon in COOH-MWCNTs-incorporated supports, which featured greater porosity, improved hydrophilicity, and lower tortuosity with finger-like pore structures [48,99,100]. It was noticeable that TFC-1 exhibited lower water flux than TFC-3 and TFC-4 under ALFS mode, despite its higher hydrophilicity compared to all other membranes (see Figure 8b). This suggested that obtaining higher overall porosity and interconnected pore structure might be more effective than hydrophilicity alone for enhancing water flux and reducing the ICP phenomenon [101,102,103]. In the ALDS mode, as illustrated by Figure 10b, the J_w_ notably increased from 4.71 LMH for TFC-0 to 9.068 LMH for TFC-4, demonstrating superior water flux performance for TFC-4. Furthermore, TFC-4 achieved a higher water flux than TFC-3 (7.80 LMH) in ALDS mode, which can be attributed to its larger pore size relative to TFC-3 (refer to Table 2 and FE-SEM bottom images in Figure 7b), facilitating improved water flow toward the DS [51,104]. Overall, all fabricated TFC membranes, in ALDS mode, exhibited greater water flux than in ALFS mode. This is because the more severe dilutive ICP in ALFS mode reduces the effective osmotic pressure driving force within the support layer [54,105]. The variations in water flux for TFC-FO membranes in ALDS mode aligned well with changes in water permeability of the corresponding membranes (Table 4). The improved water flux suggested that adding COOH-MWCNTs could significantly boost the water flux of modified TFC membranes by approximately 161% in ALFS compared to approximately 92% in ALDS modes. The increased water permeability of TFC membranes resulted from the incorporation of COOH-MWCNTs, which enhanced the porosity and hydrophilicity of membrane supports (refer to Table 2 and Figure 5b). Furthermore, this also improved the structural properties of the membrane supports to facilitate easier transport of water molecules and contributed to forming more porous, thin PA active layers (as seen in Figure 9b).

The J_s_ of all fabricated membranes, tested in both ALFS and ALDS modes, are depicted in Figure 10a and 10b, respectively. In general, enhancing the water flux of the modified TFC-FO membranes led to higher reverse solute flux, highlighting a trade-off where increased water flux corresponded to increased solute flux. As demonstrated in Figure 10a, the J_s_ of TFC membranes in ALFS mode increased from 26.94 gMH for TFC-0 to 83.10 gMH for TFC-2 (0.25 wt.% COOH-MWCNT), whereas in ALDS mode (as shown in Figure 10b), it increased from 38.048 gMH for TFC-0 to 103.47 gMH for TFC-2. Additionally, the membranes with the highest water flux, specifically TFC-3 in ALFS mode and TFC-4 in ALDS mode, exhibited moderate reverse solute fluxes of approximately 65.83 gMH and 93 gMH, respectively. Overall, the increase in the J_s_ could be ascribed to the high effective area of the support layer from the increased porosity [86,106]. Moreover, a more hydrophilic support layer can enhance both water and reverse salt flux due to enhanced “wetted porosity” [107,108]. To further assess the performance of TFC-FO membranes, it was essential to consider the specific reverse salt flux (J_s_/J_w_, g/L), which is a key measure of the membrane performance and selectivity [2,109]. A high J_s_/J_w_ ratio typically signifies low selectivity, which is generally preferred in the FO process [110]. Figure 10a,b shows the J_s_/J_w_ ratio results for all TFC membranes in both modes. In the ALFS mode, TFC-3 and TFC-4 had lower J_s_/J_w_ ratios of approximately 8.8 and 9.62 g/L, respectively, compared to TFC-0 which demonstrated a ratio of approximately 9.34 g/L. Conversely, in the ALDS mode, TFC-0 revealed the lowest J_s_/J_w_ at approximately 8.07 g/L compared to TFC-3 and TFC-4 with approximately 13.21 and 10.18 g/L, respectively. Among all membranes, TFC-2 notably showed a higher J_s_/J_w_ ratio in both ALFS and ALDS modes at approximately 16.032 and 19.34 g/L, respectively. This substantial reverse solute flux, combined with lower water flux for the TFC-2 membrane, may be ascribed to the significant impact of ICP.

### 3.5. Effect of Incorporating COOH-MWCNTs on Membrane Separation Properties

Table 4 shows the obtained values of A and B permeability coefficients, R%, and S parameters of the synthesized TFC-FO membranes. The (A) and (B) values were examined in ALFS mode due to their direct impact on water and reverse solute fluxes, whereas (S) was analyzed within the membrane substrate since it generally influences the osmotic pressure at the boundary between the active layer and the substrate in ALDS mode [58]. Simultaneously, (R%) was determined with 0.25 M NaCl in ALDS mode, utilizing a similar FO setup. The findings indicated that the modified TFC membranes exhibited greater water permeability relative to the control membrane. For instance, the water permeability of the membrane with 0.5 wt.% COOH-MWCNTs (TFC-3) was approximately 89% higher than that of the control membrane (TFC-0). The increased (A) values for the modified TFC membranes result from the decreased barrier to water diffusion within the substrate [111]. This demonstrates that enhancing the membrane support with nanomaterials boosts water permeability, considering that optimizing the support layer is typically crucial for improvement of performance of membranes [112]. As for the solute permeabilities, all modified TFC membranes exhibited a trade-off relationship and followed a comparable upward pattern as for the water permeability. Nevertheless, improving water permeability could be key to optimizing membrane performance [48]. The solute rejection rates of the modified membranes, however, were lower than that of the control membrane (TFC-0) as a result of the reduced cross-linking degree of the PA layer in the modified membranes, leading to decreased rejection performance [27]. In terms of the obtained results for the structure parameter (S), the integration of COOH-MWCNTs into the substrates of TFC membranes led to a notable decrease in (S) values compared to the control membrane. Notably, the (S) parameter dropped from 1029.31 μm for TFC-0 to 116.55 μm for TFC-3. The reduction in (S) value was likely attributed to the improved pore structure and connectivity of the membrane support in TFC-3 after incorporating COOH-MWCNTs [113]. This finding indicated that adding COOH-MWCNTs into the microstructure of the membrane support could mitigate the impact of ICP within the TFC membranes and hence enhance water permeability [114].

**Table 4:** Separation parameters of TFC-FO membranes.

Membranes	A (LMH/bar)	R^2^ (J_w_)	B (LMH)	R^2^ (J_s_)	R (%)	S (μm)	Ew%
TFC-0	0.1169	0.99	0.7884	0.99	99.60	1029.31	20.21
TFC-1	0.2078	0.95	2.1113	0.99	98.98	166.75	13.79
TFC-2	0.1578	0.94	2.1863	0.99	98.96	235	27.64
TFC-3	0.2211	0.97	2.4255	0.99	98.94	116.55	13.72
TFC-4	0.2644	0.98	2.5723	0.99	99.01	162.5	16.24

### 3.6. Fouling Resistance

**Figure 11:**
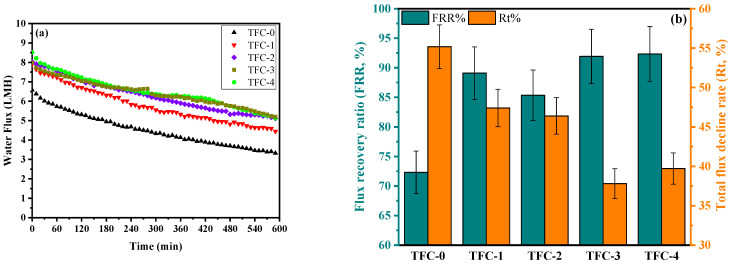
(**a**) Water flux profile during fouling experiments in FO desalination process, and (**b**) anti-fouling properties (i.e., flux recovery ratio and total reduction rate) of TFC-FO membranes. Test conditions: [FS = 50 mM NaCl, 4 mM CaCl_2_, and 500 ppm SA, DS = 2 M NaCl; Q = 250 mL/min; T = 25 ± 2 °C)].

Fouling is a significant issue impacting the membrane separation effectiveness in a variety of water treatment techniques, such as desalination of brackish and seawater, as well as wastewater purification [115]. The membrane’s potential to resist fouling is essential for maintaining long-term performance and durability in commercial-scale desalination systems. The tendency for fouling greatly influences both the effectiveness and longevity of membrane separations, as it results in the build-up of foulant substances on the surface and within the pores of TFC-FO membranes [64,65,116]. Organic fouling, as a common form of fouling, results from the adsorption of organic molecules found in natural waters and wastewater effluents. In this study, sodium alginate was chosen as a representative organic contaminant for assessing fouling, simulating typical polysaccharides, which are primary components of organic material present in wastewater and seawater [115,117]. To ensure the prolonged stability of the modified TFC-FO membranes, their resistance to fouling was tested and compared with the original TFC-FO membrane. Figure 11a illustrates the decline in water flux over a 10-h period during which the feed solution contained an elevated concentration of SA (500 ppm) along with 50 mM NaCl. To accelerate the fouling process, 4 mM CaCl_2_ was also used as a bridging agent. (FRR%) and (Rt%) values were determined for all fabricated membranes based on their fouling behavior obtained via real-time water flux monitoring, and these findings are presented in Figure 11b.

All the modified membranes exhibited a smaller reduction in water flux and a higher recovery ratio compared to the control membrane (TFC-0). As illustrated in Figure 11a, all the fabricated membranes experienced a decline in water flux during the FO process. This reduction resulted from the presence of calcium ions in the feed solution, which increased the intermolecular bonding between alginate molecules. The strengthened adhesion was caused by calcium ions forming intermolecular bridges, leading to the development of a cross-linked alginate gel layer on the membrane’s surface [118]. The resulting dense layer could act as a barrier to water molecules, causing a continuous decrease in water flux and leading to more pronounced membrane fouling [119,120]. In terms of total flux reduction (Rt%), the modified membranes outperformed the control, following the order TFC-3 > TFC-4 < TFC-2 > TFC, with reductions of 37.81%, 39.71%, 46.39%, and 47.40%, respectively, compared to TFC-0, which had a reduction of approximately 55.18% (Figure 11b). Although the TFC-1 membrane possessed the lowest contact angle, its notable total flux reduction (Rt%) compared to other modified membranes indicated that hydrophilicity alone was insufficient for anti-fouling effectiveness. The significantly high O/N ratio observed for TFC-1, as shown in Table 3, indicates an incomplete IP reaction, possibly leading to an increase in carboxylic acid groups within the PA layer. This typically results in more severe membrane contamination caused by both organic and inorganic substances [121,122,123]. Such conditions may enhance strong interactions with calcium and alginate, promoting the formation of highly structured complexes on the membrane surface [117,124]. These complexes form a cohesive fouling layer, which could have a major effect on the observed decline in water flux with TFC-1 in comparison to other modified membranes. This highlights the role of membrane surface characteristics in fouling formation and overall performance.

The fouling behavior of TFC membranes is potentially related to surface roughness and zeta potential values, with the latter governing electrostatic interactions with sodium alginate (−25 mV, see Figure S2) according to the Donnan effect theory of oppositely charged ions. The Donnan effect describes the selective distribution of ions near charged surfaces, influenced by the membrane’s surface charge and the ionic composition of the surrounding solution [56,125,126]. It is widely recognized that membranes with more negative zeta potentials and smoother surfaces typically show better resistance to fouling. However, in this study, TFC-1, despite having the smoothest surface (36.92 nm), experienced more severe fouling than TFC-2 (42.85 nm), TFC-3 (46.26 nm), and TFC-4 (56.75 nm). This unexpected result could be attributed to a cohesive fouling mechanism, where divalent ions (Ca^2+^) form bridging interactions between alginate molecules and the membrane surface. Additionally, the moderate zeta potential of TFC-1 (−18.8 mV) was less effective in repelling negatively charged foulants. In contrast, TFC-2, which had a moderately rough surface and the most negative zeta potential (−24.9 mV), exhibited good antifouling capabilities. TFC-3, with a slightly rougher surface (46.26 nm) and a moderately negative zeta potential (−14.9 mV), showed the best antifouling ability due to an optimal balance of surface roughness and charge. Although TFC-4 had greater roughness (56.75 nm) and a less negative zeta potential (−6.69 mV), it demonstrated better fouling resistance than TFC-2 and TFC-1, but was surpassed by TFC-3. Moreover, TFC-3 and TFC-4 exhibited superior water permeability and selectivity (J_s_/J_w_) compared to the other membranes, possibly resulting from their advantageous support layer structures, which partially reduced ICP and enhanced their antifouling properties [99,127]. Comparatively, TFC-0, with the highest roughness (60.39 nm) and the least negative zeta potential (−4.81 mV), displayed the weakest fouling resistance. These findings underscore the complex nature of fouling behavior, where factors such as surface roughness, zeta potential, water permeability, selectivity, and the structure of the support layer all play a crucial role in membrane anti-fouling performance. They highlight that achieving antifouling efficiency requires balancing surface roughness with zeta potential, where optimal roughness, strong electrostatic repulsion, and an improved membrane microstructure are key to superior performance. Furthermore, the sequence of fouling resistance in terms of flux recovery rate (FRR%), as depicted in Figure 11b, was as follows: TFC-4 (92.34%) > TFC-3 (91.92%) > TFC-1 (89.10%) > TFC-2 (85.36%) > TFC-0 (74.61%). Generally, the FO membrane process achieved a high FRR, despite the development of a dense yet soft and pliable alginate gel layer in FO mode, where no hydraulic pressure was applied. This suggested that the fouling layer had a loose structure that could be easily removed during the washing process [128].

### 3.7. Flux Stability During Real Seawater Test

Testing the performance of the fabricated TFC-supported membranes under real seawater conditions is also crucial for evaluating their practical applicability in FO desalination and water treatment processes [129]. Real seawater presents a more complex fouling environment, as it contains a diverse mixture of organic matter, biofoulants, and inorganic salts, leading to synergistic fouling effects that are challenging to replicate in controlled conditions [130]. This makes real seawater testing a necessary step to validate the long-term stability, anti-fouling properties and efficacy, and operational reliability of TFC membranes under practical conditions.

**Figure 12:**
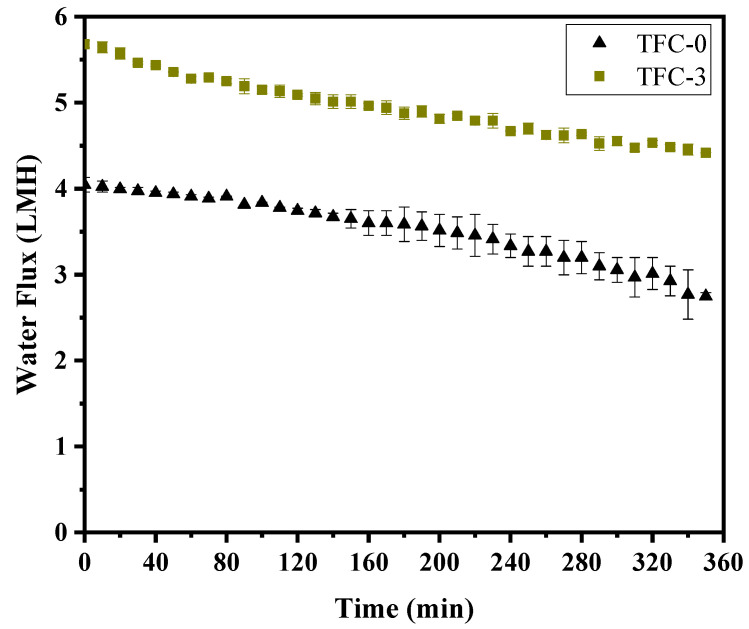
Water flux profile of control TFC-0 and optimum TFC-3 membranes over 360 min during FO process using real seawater, collected from Kuantan beach near to Teluk Chempedak, river mouth in Pahang, Malaysia.

Building on the antifouling mechanisms described in Section 3.6, Figure 12 represents flux stability findings during real seawater testing, which further validated the improved performance of the optimized TFC membrane (TFC-3). The figure compares water flux over 360 min for TFC-0 (Control) and TFC-3 membranes, tested with seawater collected near Kuantan beach, Malaysia. The compositions of the real seawater sample applied in this study are analyzed and detailed in Table S1, providing essential context for the observed results. As indicated in Figure 12, the TFC-0 membrane exhibited a significant flux decline over time, reflecting its higher susceptibility to fouling due to rougher surface texture, less negative zeta potential, and a subsidiary membrane microstructure. In contrast, the optimized TFC-3 membrane demonstrated greater flux stability, underscoring the effectiveness of its optimized properties, such as moderate surface roughness, more negative zeta potential, and balanced hydrophilicity. Mechanistic insights illustrated that the TFC-3 membrane’s optimized anti-fouling characteristics (e.g., hydrophilicity and better selectivity) reduced foulant adhesion and enhanced cleaning efficiency. Furthermore, the more negative zeta potential of the TFC-3 membrane likely repelled negatively charged foulants more effectively than the TFC-0 membrane. By tuning the zeta potential and hydrophilic properties, the optimized TFC-3 membrane achieved a balance, optimizing the Donnan effect to mitigate fouling while maintaining desirable permeability and selectivity. Consequently, the reduced flux declined in the TFC-3 membrane, which corroborated the laboratory results under controlled conditions (e.g., sodium alginate as a foulant). The high flux recovery ratio (FRR) of the TFC-3 membrane observed during testing with synthetic foulants translated to better real-world performance, reflecting the robustness of its design. Despite these observations, the findings emphasized that achieving antifouling efficiency remains a multifactorial challenge, with factors such as membrane microstructure and chemical interactions playing critical roles.

Table 5 illustrates a comparison of the present work with existing literature, which covers membrane performance properties, including J_w_, J_s_, R%, S parameter, and antifouling property. Herein, the performance of the PA-TFC membrane modified with 0.5 wt.% COOH-MWCNTs in the PES support layer was comprehensively compared with previous studies employing nanomaterials in FO membranes. The present study achieved a J_w_ of 7.48 LMH in ALFS mode, which was lower than some reported values in the literature. For instance, Sirinupong et al. [24] incorporated 0.5 wt.% graphene oxide (GO) into a PA/PSF membrane and achieved ~11 LMH, whereas Li et al. [31] used 0.15 wt.% GO and reported a J_w_ of 28.5 LMH. Notably, Yu et al. [131] employed 3 wt.% sulfonated graphene oxide (SGO), achieving the highest flux of 38.12 LMH. However, the J_w_ in the present study was comparable to that of Wang et al. [29] and Choi et al. [48], who reported ~12 and 11.98 LMH, respectively, using functionalized MWCNTs and functionalized carboxyl carbon nanofibers (f-CNFs). Further, the J_s_/J_w_ ratio in the present work (8.80 g/L) was significantly higher than most reported values, except for Aziz et al. [54] (J_s_/J_w_ 0.01297 g/L) and Sirinupong et al. [24] (J_s_/J_w_ 0.318 g/L). This suggested that while the present work enhanced water transport, it may slightly trade off selectivity due to the hydrophilic properties of COOH-MWCNTs altering the PA layer’s structure. On a separate point, a key strength of the present work was its high solute rejection (98.94%), outperforming most studies, including Choi et al. [48] (90.46%), Li et al. [31] (90.5%), and Hadadpour et al. [109] (91.3%). Comparably, only Aziz et al. [54] reported a slightly higher R% (97.89%) with protonated carbon nitride (pCN)-modified PA/PSF membranes. On the other hand, the S parameter of 116.55 μm in the present work was relatively low compared to that reported by Xu et al. [105] (387 μm) and Tavakol et al. [28] (788.2 μm), indicating a reduced ICP effect. A lower S value correlated with improved osmotic driving force, thus supporting high flux and efficiency in FO applications. The present study demonstrated high antifouling properties, consistent with those reported by Choi et al. [48] and Hadadpour et al. [109], who also used carbon-based nanomaterials to enhance hydrophilicity and mitigate fouling. The incorporation of COOH-MWCNTs resulted in electrostatic repulsion against foulants, aligning with previous findings from Wang et al. [29]. Compared to the existing literature, the present study presented a balanced trade-off between water permeability and solute rejection. While the J_s_/J_w_ ratio remained within acceptable limits, the study achieved competitive water flux values and exceptional solute rejection, alongside improved antifouling performance. This suggests that COOH-MWCNTs in the PES support layer could provide significant benefits in optimizing FO membrane performance while maintaining a high degree of selectivity and stability. However, it is important to note that the intrinsic trade-off between permeability and selectivity remains a fundamental limitation in membrane design, as enhancing water flux often leads to increased solute passage, and, conversely, improving selectivity typically reduces water flux. Placing our results within this widely observed ‘upper-bound’ behavior highlights the critical importance of achieving low J_s_/J_w_ while sustaining high flux, a challenge that recent research has sought to address through strategies such as isoporous architectures, mixed-matrix, and aquaporin membranes [132]. Moreover, the large-scale manufacturing of TFC-FO membranes presents considerable challenges for commercial applications, primarily due to their relatively high production costs, which limit affordability. Nevertheless, TFC-FO membranes incorporating nanomaterials within either the support or active layers can be fabricated using processes that are relatively straightforward and compatible with existing commercial RO membrane production lines, requiring only minor modifications. This compatibility provides a viable pathway for scaling up the technology and enhancing its potential for widespread commercialization [9,133,134].

## 4. Conclusions

This study successfully integrated COOH-MWCNTs into the PES support layer during the fabrication of TFC-FO membranes. With a concentration range of 0.1–0.75 wt.%, the modified membranes exhibited notable improvements in hydrophilicity, surface porosity, total porosity, and both mechanical and thermal properties. Even at low concentrations, COOH-MWCNTs significantly influenced the microstructure and morphology of the support layer, encouraging the development of a finger-like structure and lowering the structural parameter (S-value) of the membrane. Additionally, COOH-MWCNTs affected the formation of the PA active layer, enhancing key characteristics such as hydrophilicity, surface charge, and roughness. While water permeability showed a significant increase, solute permeability also rose, emphasizing the inherent balance between water flux and solute rejection. Despite this trade-off, the modified membranes demonstrated enhanced stability. The observed decrease in solute rejection was linked to a reduced degree of cross-linking within the PA layer, a factor known to impact solute selectivity in TFC membranes.

Among the membranes evaluated, the TFC membrane containing 0.5 wt.% COOH-MWCNTs (TFC-3) displayed the highest performance in the ALFS mode. Structural analysis confirmed that incorporating COOH-MWCNTs effectively minimized ICP, as indicated by the substantial reduction in the S-value for TFC-3 (1165.55 µm) compared to the unmodified membrane (TFC-0 at 1029.31 µm). Furthermore, dynamic fouling tests revealed that TFC-3 exhibited a slower flux decline and a more rapid flux recovery than the control membrane, reinforcing its potential for real-world water treatment applications. Interestingly, membranes containing 0.75 wt.% COOH-MWCNTs displayed performance levels similar to those with 0.5 wt.% loading, suggesting that higher concentrations may not yield considerable additional benefits in membrane efficiency. This finding underscores the importance of balancing nanomaterial concentration with optimal membrane performance. Additionally, our future work is set to further investigate the long-term durability and effectiveness of these membranes under various real seawater conditions, as well as examine how different functionalization techniques impact MWCNT incorporation. Additionally, further exploration of the relationship between PA layer cross-linking density and its influence on solute rejection, alongside the development of hybrid membranes incorporating other nanomaterials, could lead to enhanced membrane performance and antifouling properties.

## Figures and Tables

**Table 1:** Composition of substrate doping solutions.

Substrate	Formulation of Dope Solution (PES/PVP/CNTs, wt.%)
MW-S0	15/5/0
MW-S1	15/5/0.1
MW-S2	15/5/0.25
MW-S3	15/5/0.5
MW-S4	15/5/0.75

**Table 2:** Porosity, pore size and thickness of membrane supports.

Membranes Support	Porosity (By Gravimetric Method) (%)	Pore Size (By Guerout-Elford-Ferry Theory) (nm)	Thickness (By FE-SEM) (μm)
MW-S0	66.45	111.90	214.16
MW-S1	69.90	74.54	131.60
MW-S2	70.18	59.82	142.29
MW-S3	73	58.03	129.61
MW-S4	72.43	79.77	128.68

**Table 3:** Surface chemistry analysis of PA-TFC membranes measured by XPS.

Membranes	C (%)	N (%)	O (%)	O/N
TFC-0	75	11.3	13.5	1.194
TFC-1	74.1	3.3	18.1	5.48
TFC-2	71	10.7	17.8	1.663
TFC-3	75.1	7.2	16.6	2.305
TFC-4	74.7	6.7	18.6	2.776

**Table 5:** Comparison of FO performance (ALFS mode) of PA-TFC membranes with nanomaterial-modified support.

Active Layer Support Layer	Optimum Loading	J_w_ (LMH)	J_s_/J_w_ (g/L)	R (%)	S (μm)	FS DS	Anti-Fouling Performance	Ref.
PA	2 wt.% COOH-MWCNTs	~12	-	~95% *	2042	0.01 M NaCl	-	[29]
PES						2.0 M glucose		
PA	0.5 wt.% fMWCNTs	11.98	0.6427	90.46%	387	DI water	High	[48]
PES						0.6 M NaCl		
PA	0.5 wt.% GO	~11	~0.318	90.1%	420	DI water	-	[24]
PSF						2.0 M NaCl		
PA	4 wt.% CNPs	14	0.357	-	-	DI water	High	[105]
PES						1.0 M NaCl		
PA	0.15 wt.% GO	28.5	0.421	90.5%	-	DI water	-	[31]
PSF						2.0 M NaCl		
PA	0.5 wt.% pCN	4.24	0.01297	97.89%	2960	DI water	-	[53]
PSF						1.0 M NaCl		
PA	0.3 wt.% f-CNFs	13.08	0.24	94.5%	788.2	DI water	-	[28]
PSF						1.0 M NaCl		
PA	10 wt.% HCDs	15.47	0.1874	94.4%	188	DI water	-	[113]
PAN						1.0 M NaCl		
PA	0.5 wt.% GNPs	19.97	0.5172	95.50%	449	DI water	-	[81]
PSF						1.0 M NaCl		
PA	0.5 wt.% CNM	12.08	0.2458	91.3%	883.4	DI water	High	[109]
PES						1.0 M NaCl		
PA	3 wt.% SGO	38.12	~0.0041	-	-	DI water	-	[131]
PSF						2.0 M NaCl		
PA	0.5 wt.% COOH-MWCNTs	7.48	8.80	98.94% *	116.5	DI water	High	Present work
PES						1.0 M NaCl		

* Solute rejection measured by FO process.

## Data Availability

The data presented in this study are available on request from the corresponding authors.

## Supplementary Materials

The following supporting information can be downloaded at: https://www.mdpi.com/article/10.3390/membranes15080240/s1, Figure S1: FO performance of TFC membranes (**a**,**b**) Jw and Js under AL-FS mode; (**c**,**d**) Jw and Js under AL-DS mode. Operating conditions: [FS: pure water, DS = 0.25–1.0 M NaCl; Q = 250 mL/min; T = 25 ± 2 °C, Am= 42 cm2]; Figure S2: Zeta potential of mode foulant. Test condition: [FS: 50 mM NaCl, 4 mM CaCl2 and 500 ppm SA, T = 25 ± 2 °C]; Table S1: Seawater compositional analysis.

## Abbreviations

The following abbreviations are used in this manuscript:

AFM	Atomic Force Microscopy
ALDS	Active Layer Facing Draw Solution Mode
ALFS	Active Layer Facing Feed Solution Mode
BET	Brunauer-Emmett-Teller
CA	Contact Angle
CNM	Charcoal-based Carbon Nanomaterial
CNPs	Carbon Nano-Particles
COOH-MWCNTs	Carboxyl-Functionalized Multiwalled Carbon Nanotubes
DI	Deionized Water
DS	Draw Solution
f-CNFs	Functionalized Carboxyl Carbon Nanofibers
fMWCNTs	Functionalized Multi-Wall Carbon Nanotubes
FE-SEM	Field Emission Scanning Electron Microscopy
FO	Forward Osmosis
FRR	Flux Recovery Ratio
FS	Feed Solution
FTIR	Fourier-Transform Infrared Spectroscopy
GNPs	Graphene Nanoplatelets
GO	Graphene Oxide
HCDs	Hydrophobic Carbon Dots
ICP	Internal Concentration Polarization
IP	Interfacial Polymerization
J_s_	Reverse Solute Flux
J_w_	Water Flux
LMH	Liters per Square Meter per Hour
MMM	Mixed Matrix Membrane
MPD	m-Phenylenediamine
MWCNTs	Multiwalled Carbon Nanotubes
NaCl	Sodium Chloride
NMP	N-Methyl-2-pyrrolidone
PA	Polyamide
pCN	Protonated Carbon Nitride
PEG	Polyethylene Glycol
PES	Polyethersulfone
pDA	Polydopamine
PSf	Polysulfone
PVA	Polyvinyl Alcohol
PVP	Polyvinylpyrrolidone
RO	Reverse Osmosis
RT	Room Temperature
Rt	Total Flux Decline Ratio
SA	Sodium Alginate
SGO	Sulfonated Graphene Oxide
SRSF	Specific Reverse Solute Flux
σ	Tensile Strength
TFC	Thin-Film Composite
TGA	Thermogravimetric Analysis
TMC	Trimesoyl Chloride
WCA	Water Contact Angle
XPS	X-ray Photoelectron Spectroscopy
XRD	X-ray Diffraction
ZP	Zeta Potential
