# Supplementary material for: Antifouling and Desalination Enhancement of Forward Osmosis-Based Thin Film Composite Membranes via Functionalized Multiwalled Carbon Nanotubes Mixed Matrix Polyethersulfone Substrate
Source: Membranes (Basel). 2025 Aug 8;15(8):240. doi: 10.3390/membranes15080240 (PMC12388661; doi:10.3390/membranes15080240)
Supplement: Supplementary file 1 [file membranes-15-00240-s001.zip › membranes-3765189-supplementary.pdf]

Antifouling and desalination enhancement of forward osmosis-based thin film composite membranes *via* functionalized multiwalled carbon nanotubes mixed matrix polyethersulfone substrate

Hamza E. Almansouri [a,b*], Mohamed Edokali [c], Mazrul N. Abu Seman [a,d*], Ellora Priscille Ndia Ntone [a], Che Ku Mohammad Faizal Che Ku Yahya [a], Abdul Wahab Mohammad [e*]

[a] *Faculty of Chemical and Process Engineering Technology, Universiti Malaysia Pahang Al-Sultan Abdullah, Lebuhraya Persiaran Tun Khalil Yaakob, Kuantan, Gambang 26300, Pahang, Malaysia.*

[b] *Chemical Engineering Department, Faculty of Engineering, University of Benghazi, Benghazi, Libya.*

[c] *School of Chemical and Process Engineering, University of Leeds, Leeds, LS2 9JT, United Kingdom.*

[d] *Centre for Sustainability of Mineral and Resource Recovery Technology (SMaRRT), Universiti Malaysia Pahang Al-Sultan Abdullah, Lebuh Persiaran Tun Khalil Yaakob, Kuantan, Pahang, Gambang 26300, Malaysia.*

[e] *Chemical and Water Desalination Program, College of Engineering, University of Sharjah, Sharjah 27272, United Arab Emirates.*

[*] *Corresponding authors:*

- E-mail address: mazrul@umpsa.edu.my (*M. Abu Seman*).
- E-mail address: hamza.almansouri@uob.edu.ly (*H. Almansouri*).

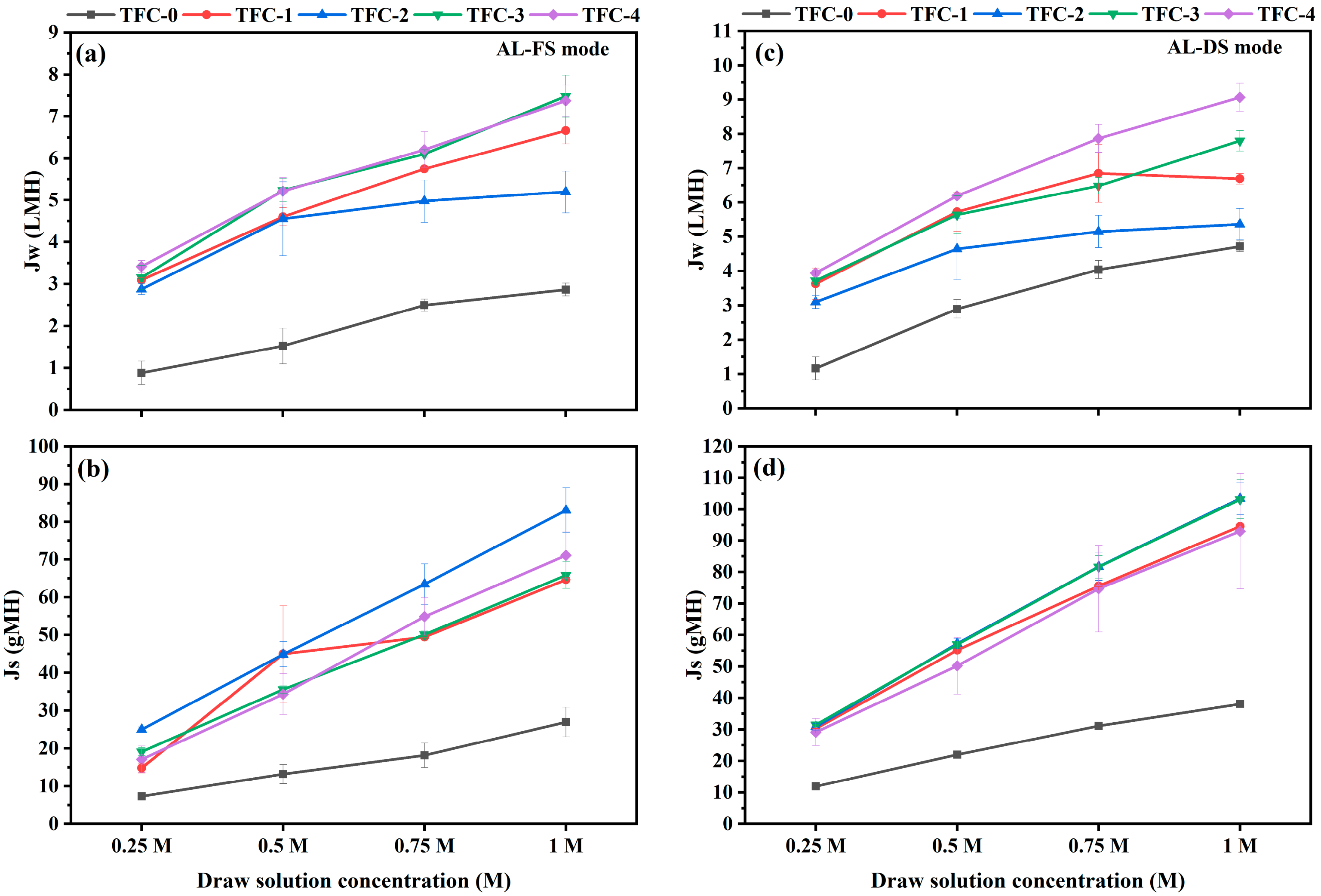

Fig. S1. FO performance of TFC membranes **(a, b)** Jw and Js under AL-FS mode; **(c, d)** Jw and Js under AL-DS mode. Operating conditions: [*FS: pure water, DS=0.25-1.0 M NaCl; Q=250 ml/min; T=25 ± 2 °C, Am= 42 cm*^{2}].

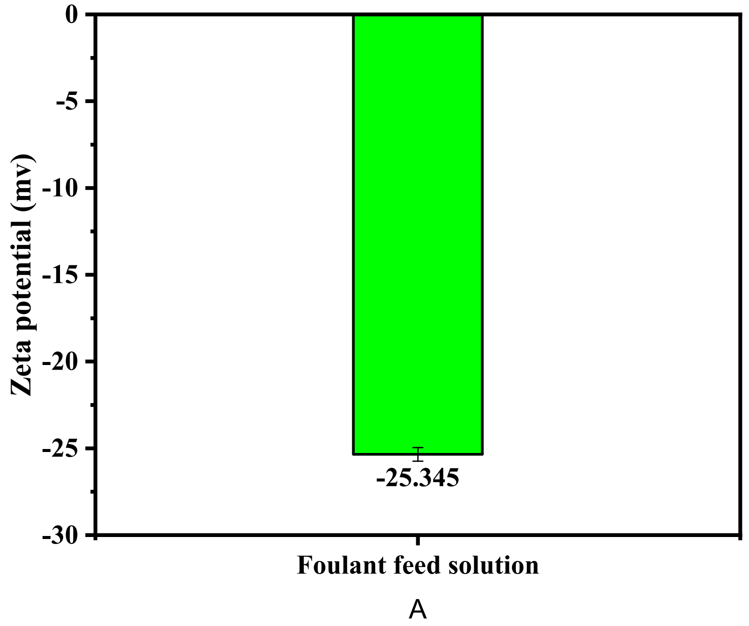

Fig. S2. Zeta potential of mode foulant. Test condition: [*FS: 50 mM NaCl, 4 mM* $CaCl_2$ *and 500 ppm SA, T=25 ± 2 °C*].

Table S1. Seawater compositional analysis

No	Parameter	Results	Unit	Test Method
1.	Sodium (Na)	5702.5	ppm	In-house Method based on APHA 3210
2.	Magnesium (Mg)	677.02	ppm	In-house Method based on APHA 3210
3.	Potassium (K)	216.32	ppm	In-house Method based on APHA 3210
4.	Calcium (Ca)	48.055	ppm	In-house Method based on APHA 3210
5.	Silicon (Si)	20.189	ppm	In-house Method based on APHA 3210
6.	pH	7.6 @ 25.0 °C	-	APHA 4500^{+}H
7.	Turbidity	7.68	NTU	APHA 2130
8.	Conductivity	39.36	ms/cm	In-house Method using Conductivity Meter
9.	Salinity	25.15	psu	In-house Method using Conductivity Meter
10.	Alkalinity	10	mg/L	In-house based on APHA 4500
11.	Total Hardness	1,730	mg/L	In-house based on APHA 4500
12.	Total Organic Carbon	195	mg/L	Merck method 14878
13.	Total Suspended Solid	89	mg/L	APHA 2540 D
14.	Total Dissolved Solids	12.95	ppt	APHA 2540
